# The liver protection of propylene glycol alginate sodium sulfate preconditioning against ischemia reperfusion injury: focusing MAPK pathway activity

**DOI:** 10.1038/s41598-017-15521-3

**Published:** 2017-11-09

**Authors:** Shizan Xu, Peiqin Niu, Kan Chen, Yujing Xia, Qiang Yu, Ning Liu, Jingjing Li, Sainan Li, Liwei Wu, Jiao Feng, Wenwen Wang, Xiya Lu, Tong Liu, Fan Wang, Weiqi Dai, Xiaoming Fan, Wenhui Mo, Ling Xu, Chuanyong Guo

**Affiliations:** 1 Department of Gastroenterology, Shanghai Tenth People’s Hospital, Tongji University School of Medicine, Shanghai, 200072 China; 2 Department of Gastroenterology, Shanghai Tenth People’s Hospital, School of Clinical Medicine of Nanjing Medical University, Shanghai, 200072 China; 3 Department of Oncology, Shanghai General Hospital, Shanghai Jiaotong University School of Medicine, Shanghai, 200080 China; 4 Department of Gastroenterology, Zhongshan Hospital of Fudan University, Shanghai 200032, China; Shanghai Institute of Liver Diseases, Zhongshan Hospital of Fudan University, Shanghai, 200032 China; 5 Department of Gastroenterology, Jinshan Hospital of Fudan University, Jinshan, Shanghai, 201508 China; 6 Department of Gastroenterology, Minhang Hospital, Fudan University, Shanghai, 201100 China; 7 Department of Gastroenterology, Shanghai Tongren Hospital, Shanghai Jiaotong University School of Medicine, Shanghai, 200336 China

## Abstract

Hepatic ischemia reperfusion (IR) injury contributes to the morbidity and mortality associated with liver surgery. This study investigated the protective function and mechanism of propylene glycol alginate sodium sulfate (PSS), a sulfated polysaccharide, in a mouse hepatic IR injury model. PSS (25 or 50 mg/kg) or saline were injected intraperitoneally to male Balb/c mice 1 h before 45 min of 70% warm hepatic ischemia and 2, 8, and 24 h of reperfusion. Serum and liver tissue samples were collected for evaluation of hepatocellular damage, liver histology, and assay of inflammatory cytokines, apoptosis- and autophagy-related proteins, and proteins in the mitogen-activated protein kinase (MAPKs). Histological injury and release of transaminases, and inflammatory cytokine production were significantly reduced by PSS pretreatment. The expression of apoptosis- and autophagy-related proteins, and the activation of MAPK signal, including jun N-terminal kinase (JNK), extracellular signal-regulated kinase (ERK), and P38 were all affected by PSS treatment compared with IR model controls. PSS protected the liver from IR injury by suppressing the MAPK signaling and down-regulating inflammation, apoptosis, and autophagy.

## Introduction

Hepatic ischemia reperfusion (IR) injury, a reaction to the sudden re-establishment of blood supply following a period of hypoxia, is a clinical consequence of liver transplantation, hepatic resection, severe trauma, and hemorrhagic shock^1,2^. Reperfusion is ultimately beneficial, but hepatic IR injury is the main cause of graft rejection, dysfunction, and even failure after liver transplantation and resection^3^. Propylene glycol alginate sodium sulfate (PSS), is a heparinoid compound prepared by sulfation of low-molecular weight alginate isolated from brown algae. It has been used in China to treat hyperlipidemia and ischemic cardio-cerebrovascular diseases for nearly 30 years^4,5^. Pharmacologic agents had been widely investigated in animal models to study the protective functions against hepatic IR injury. Dalteparin, a low-molecular weight heparin can attenuate inflammatory responses and reduce hepatic IR injury^6^. PSS has been shown to protect against IR injury of the heart^7^, but it has not been evaluated in a model of hepatic IR injury.

IR injury is usually associated with sterile inflammation^8^. Activation of Kupffer cells promotes the production and release of pro-inflammatory cytokines, including tumor necrosis factor (TNF)-α, interleukin (IL)-1β, and interferon (IFN)-γ^9–11^, Leukocyte accumulation and sequestration of neutrophils and platelets in liver tissue are also characteristic events in hepatic IR injury^12^. As a heparinoid, PSS has anti-inflammatory and anticoagulant activity^13^ that are likely to protect against against IR injury. Cellular signaling that is activated in hepatic IR injury includes members of the mitogen-activated protein kinase (MAPKs) family^2,7,14^, and inhibition of the MAPKs pathway is known to be protective. Both apoptosis and necrosis occur *in vivo* with hepatic IR injury^15^. In apoptosis, programmed cell death is initiated by either extrinsic or intrinsic pathways that activate caspases such as caspase 3 and 9^16–18^. Autophagy is another form of programmed cell death that occurs with hepatic IR injury. The function of autophagy in IR injury is not clear, with some studies reporting an increase in autophagy proteins^19^ and others reporting a decrease^20^. The study objective was to evaluate the protective effectiveness of PSS pretreatment in a mouse model of warm hepatic IR injury. The underlying mechanism of PSS activity was also studied.

## Results

### PSS treatment did not affect liver function

**Figure 1:**
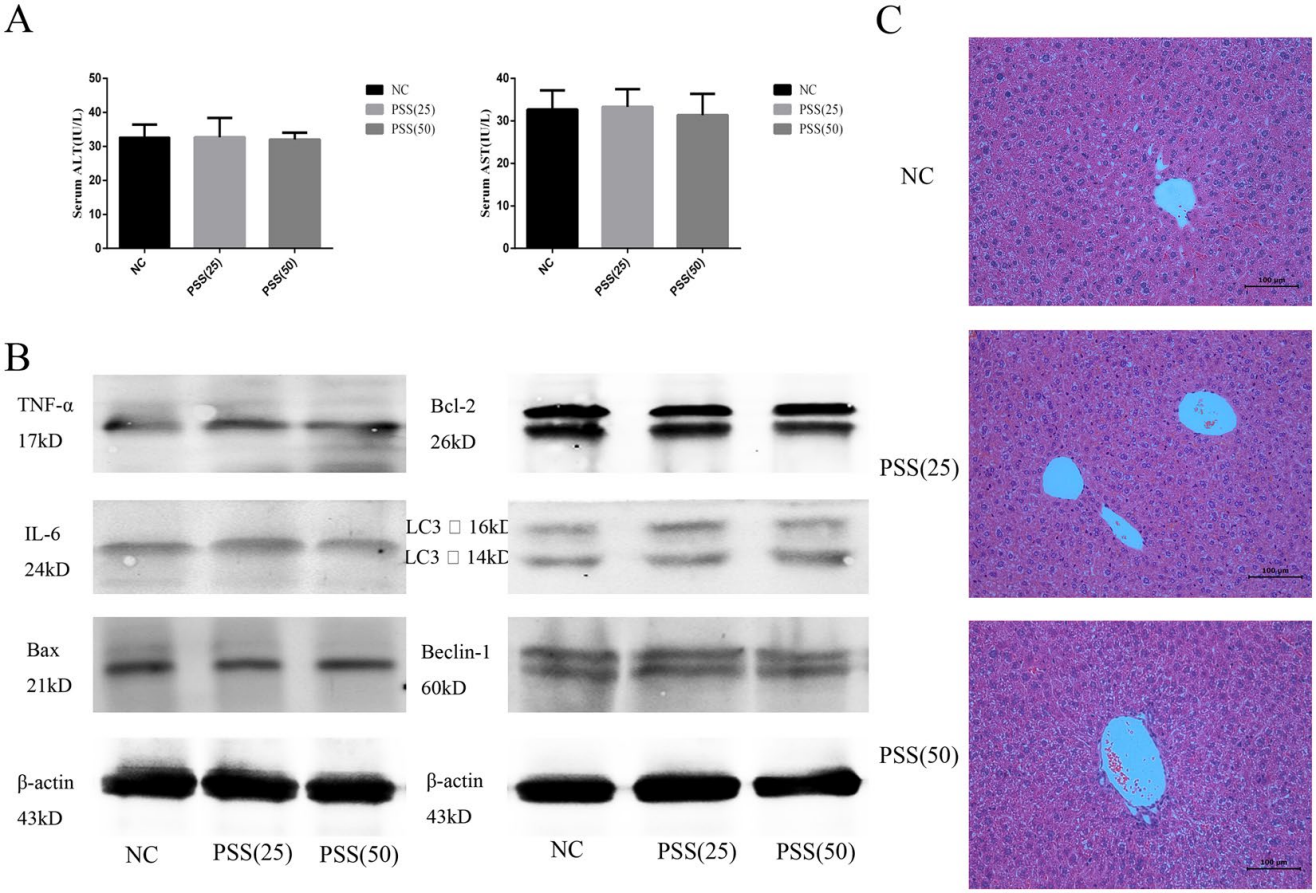
PSS (25 or 50 mg/kg) had effects on liver function as shown by (**A**) the serum ALT and AST levels in the NC, PSS (25), and PSS (50) groups 24 h after treatment. Data are means ± SD (n = 6, P > 0.05); (**B**) TNF-α, IL-6, Bax, Bcl-2, LC3, or Beclin-1 protein expression; or (**C**) liver histology following HE staining (Original magnification, ×200).

The toxicity of PSS was evaluated by changes in liver enzymes, expression of some inflammation, apoptosis, and autophagy proteins, and pathological examination. As shown in Fig. 1A, no statistically significant differences were observed in the serum ALT and AST levels in the NC group and the two PSS-treated groups. Only small differences in TNF-α, IL-6, Bax, Bcl-2, LC3, and Beclin-1 expression were seen (Fig. 1B), and HE-stained sections of liver tissue from the three groups had no obvious necrosis (Fig. 1C).

### PSS treatment attenuates IR-induced hepatocellular injury and hepatic pathological damages

**Figure 2:**
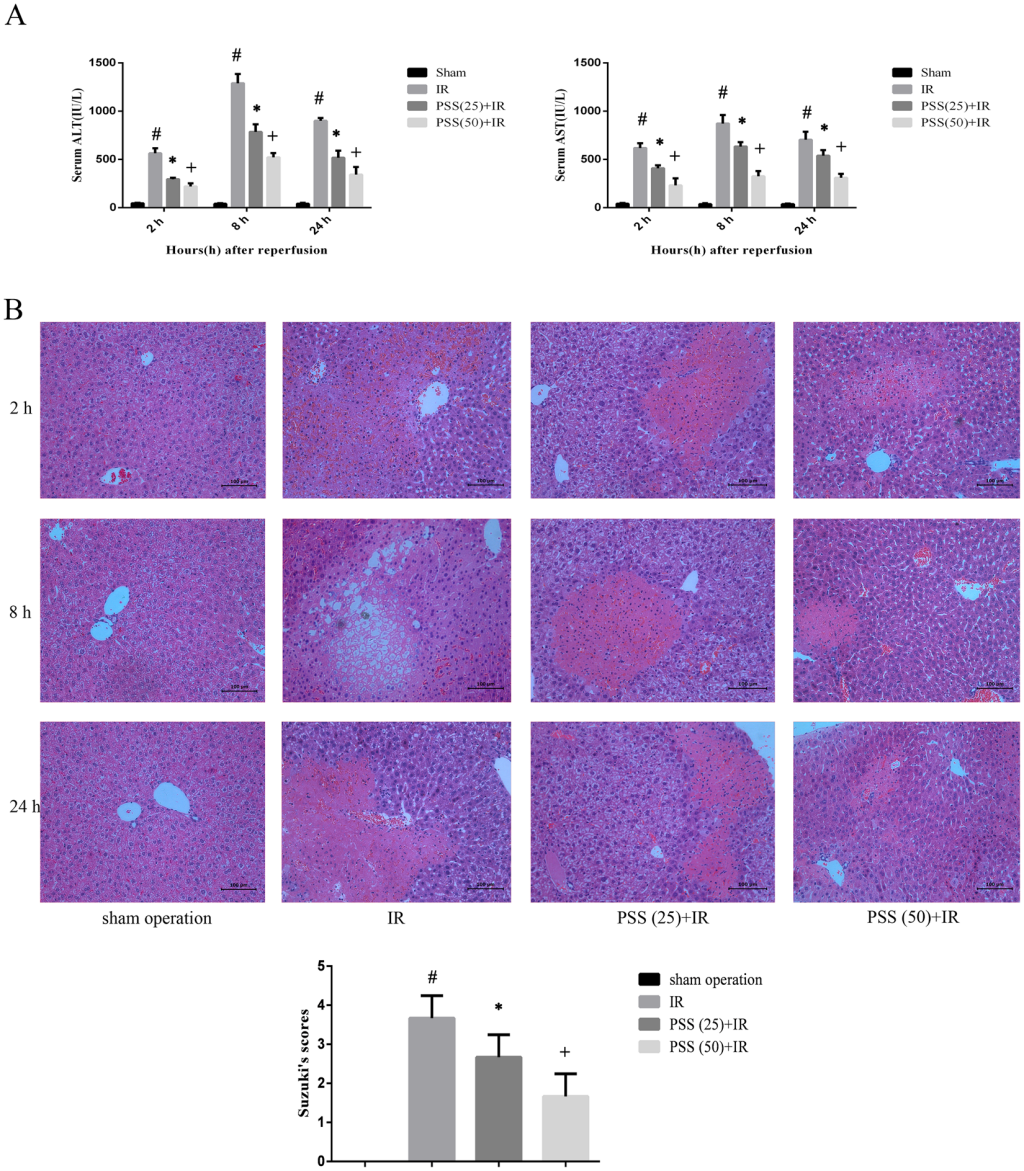
Assessment of PSS treatment in IR-induced liver injury showing (**A**) serum ALT and AST in the sham operation, IR, PSS (25) +IR, and PSS (50) +IR groups. Data are means ± SD (n = 6) ^#^P < 0.05 IR group vs. sham operation group, *P < 0.05 PSS (25) +IR group vs. IR group, ^+^P < 0.05 PSS (50) +IR group vs. IR group. (**B**) the pathological changes in liver tissue (HE staining, original magnification ×200). Severity of liver injury at 8 h was scored using the Suzuki criteria. Data are means ± SD (n = 6) ^#^P < 0.05 IR group vs. sham operation group, *P < 0.05 PSS (25) +IR group vs. IR group, ^+^P < 0.05 PSS (50) +IR group vs. IR group.

Serum ALT and AST were assayed 2, 8, and 24 h after reperfusion. In Fig. 2A, ALT and AST were significantly higher in IR-group mice than in those in the sham operation, consistent with severe injury induced by hepatic IR. ALT and AST were significantly lower in the PSS-treated groups than in the IR group, consistent with attenuation of IR-induced hepatocellular injury. The assessment of HE staining was consistent with the serum assays. Severe necrosis, sinusoidal congestion, and infiltration of inflammatory cells were seen in mice in the IR group, whereas significantly less pathological evidence of injury was noted in the PSS-treated groups (Fig. 2B). The Suzuki criteria scores were significantly lower in the PSS-treated groups than in the IR group, reflecting less severe pathological damage.

### PSS treatment prevents IR-induced inflammatory responses

**Figure 3:**
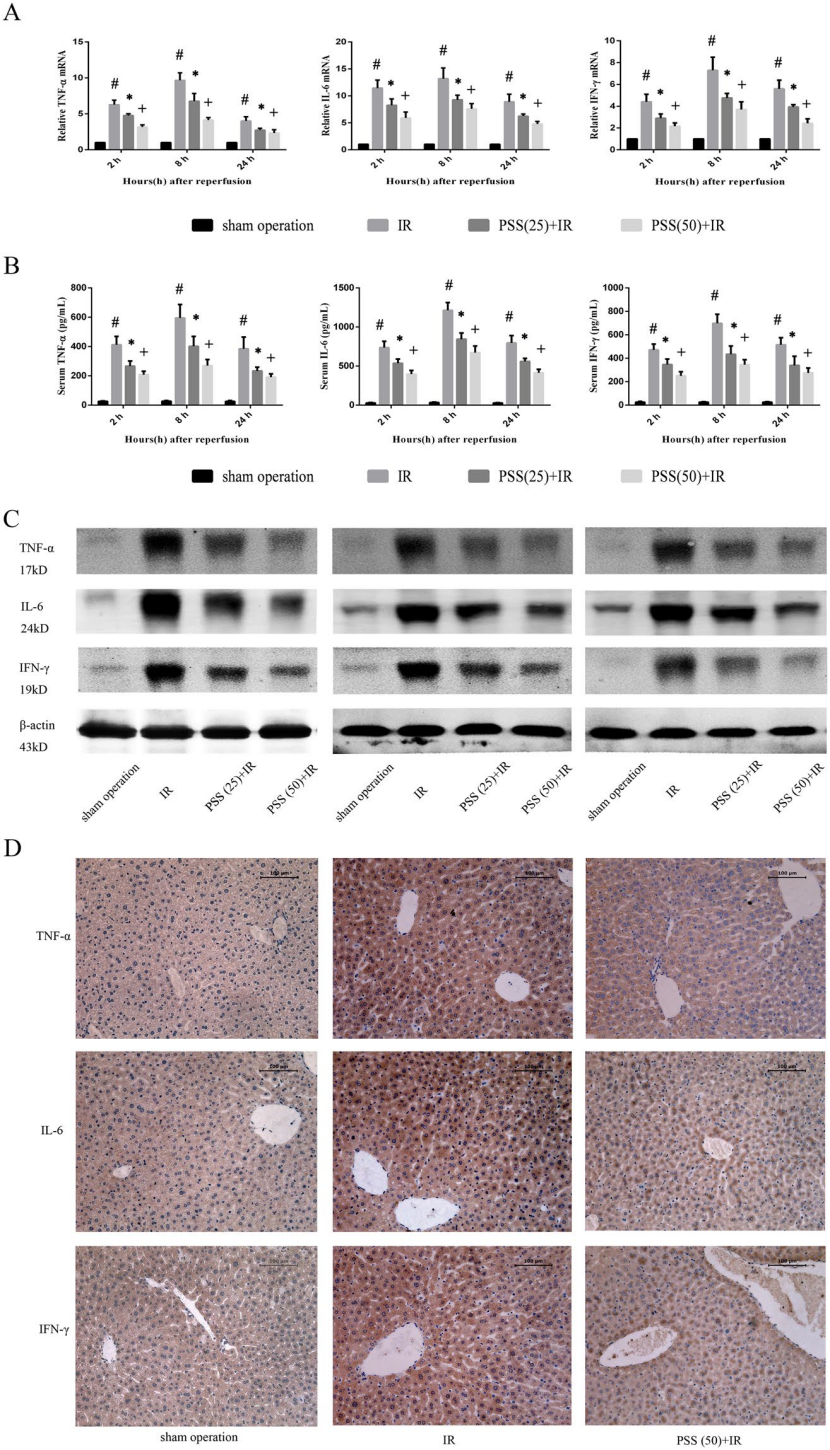
Anti-inflammatory activity of PSS in IR injury as shown by (**A**) expression of TNF-α, IL-6, and IFN-γ mRNA in liver tissue assayed by RT-PCR. Data are means ± SD (n = 6) ^#^P < 0.05 IR group vs. sham operation group, *P < 0.05 PSS (25) +IR group vs. IR group, ^+^P < 0.05 PSS (50) +IR group vs. IR group. (**B**) serum TNF-α, IL-6, and IFN-γ levels by ELISA. Data are means ± SD (n = 6) ^#^P < 0.05 IR group vs. sham operation group, *P < 0.05 PSS (25) +IR group vs. IR group, ^+^P < 0.05 PSS (50) +IR group vs. IR group. (**C**) expression of TNF-α, IL-6, and IFN-γ protein in liver tissue assayed by western blot. (**D**) TNF-α, IL-6, and IFN-γ immunostaining of liver tissue sections from sham operation, IR, PSS (25) +IR, and PSS (50) +IR groups 8 h after IR. Representative sections from six mice (Original magnification, ×200).

Expression of TNF-α, IL-6, and IFN-γ mRNA in liver samples 2, 8, and 24 h after reperfusion was assayed by quantitative RT-PCR (Fig. 3A) and found to be significantly lower in the PSS-treated groups than in the IR group. Serum TNF-α, IL-6, and IFN-γ levels were slightly increased in the sham operation group, but were significantly increased in the mice with hepatic IR. PSS pretreatment prevented the production and release of these cytokines (Fig. 3B). The western blot assay and immunohistochemical staining results for the cytokines expression consist with those obtained with RT-PCR and ELISA (Fig. 3C,D).

### PSS treatment inhibited IR-induced apoptosis

**Figure 4:**
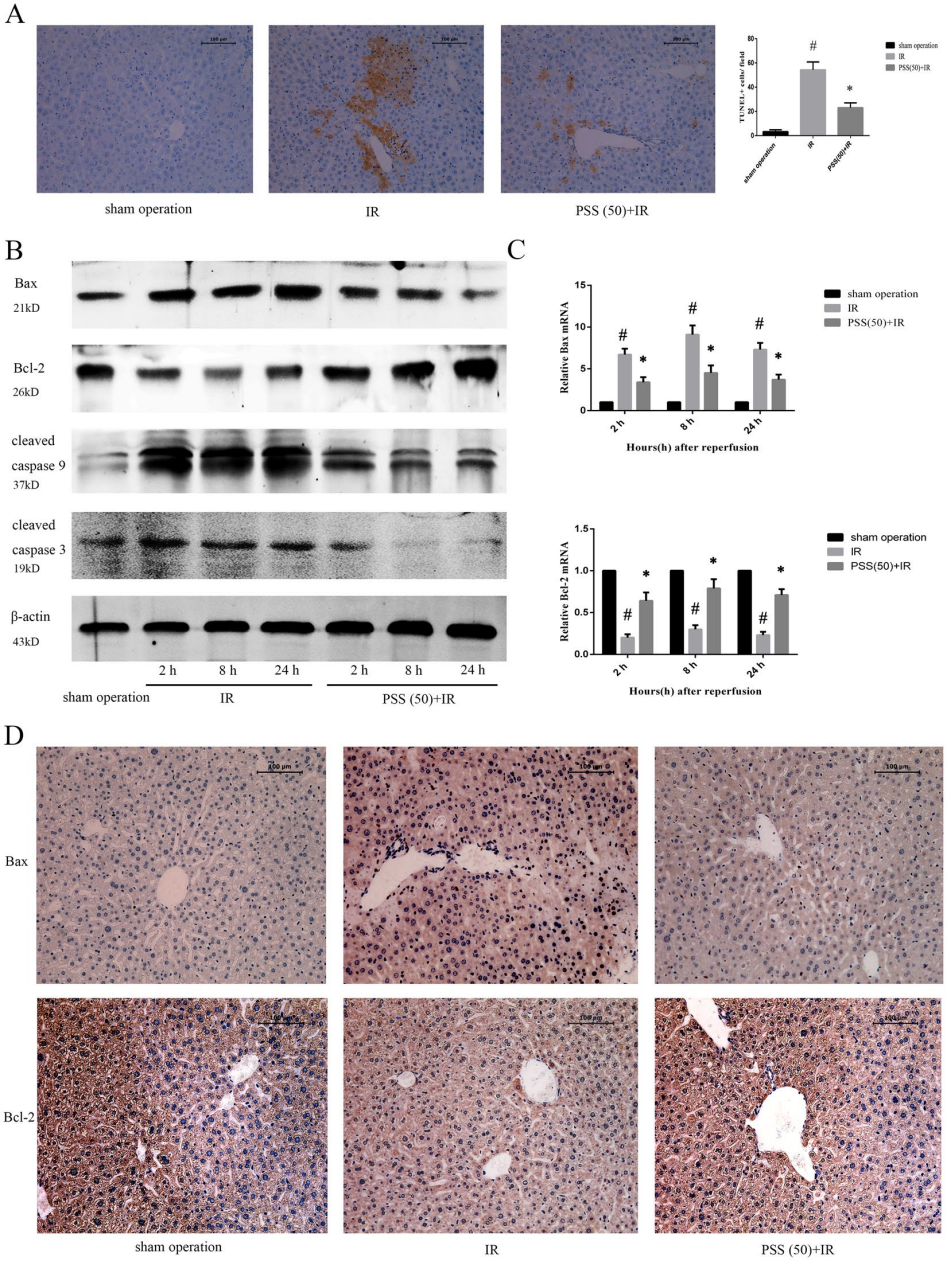
Anti-apoptotic activity of PSS treatment in hepatic IR injury as shown by (**A**) hepatocellular apoptosis 8 h after IR assayed by TUNEL staining. Both necrosis and apoptosis were found and the apoptotic cells with brown nuclei were counted. Data are means ± SD (n = 6). ^#^P < 0.05 IR group vs. sham operation group, *P < 0.05 PSS (50) +IR group vs. IR group). (**B**) expression of Bax, Bcl-2, and cleaved caspase 3 and 9 assayed by western blot. (**C**) expression of Bax and Bcl-2 mRNA was assayed by RT-PCR. Data are means ± SD (n = 6) ^#^P < 0.05 IR group vs. sham operation group, *P < 0.05 PSS (50) +IR group vs. IR group. (**D**) Bax and Bcl-2 immunostaining in liver tissue from sham operated mice, IR, and PSS (50) +IR group 8 h after IR. Representative sections from six mice (Original magnification, ×200).

Apoptosis occurring in liver tissue after IR and was evaluated using the TUNEL assay (Fig. 4A). Apoptosis was rarely observed in tissue from mice in the sham operation group, but the rate was significantly increased after IR. As expected, hepatocyte apoptosis was significantly reduced by PSS pretreatment, and the results of western blot and quantitative RT-PCR assays of apoptosis-related proteins and mRNA, including Bax, Bcl-2, and cleaved caspase 3 and 9, were consistent with the TUNEL assay results. As shown in Fig. 4B & C, Bax and cleaved caspase 3 and 9 significantly increased after IR, and the increase was prevented by PSS treatment. Bcl-2 expression was markedly decreased in IR group mice, and was promoted by PSS treatment. Immunohistochemical staining of Bax and Bcl-2 in liver tissue indicated the same changes in protein expression as the other assays (Fig. 4D).

### PSS treatment reduced IR-induced autophagy

**Figure 5:**
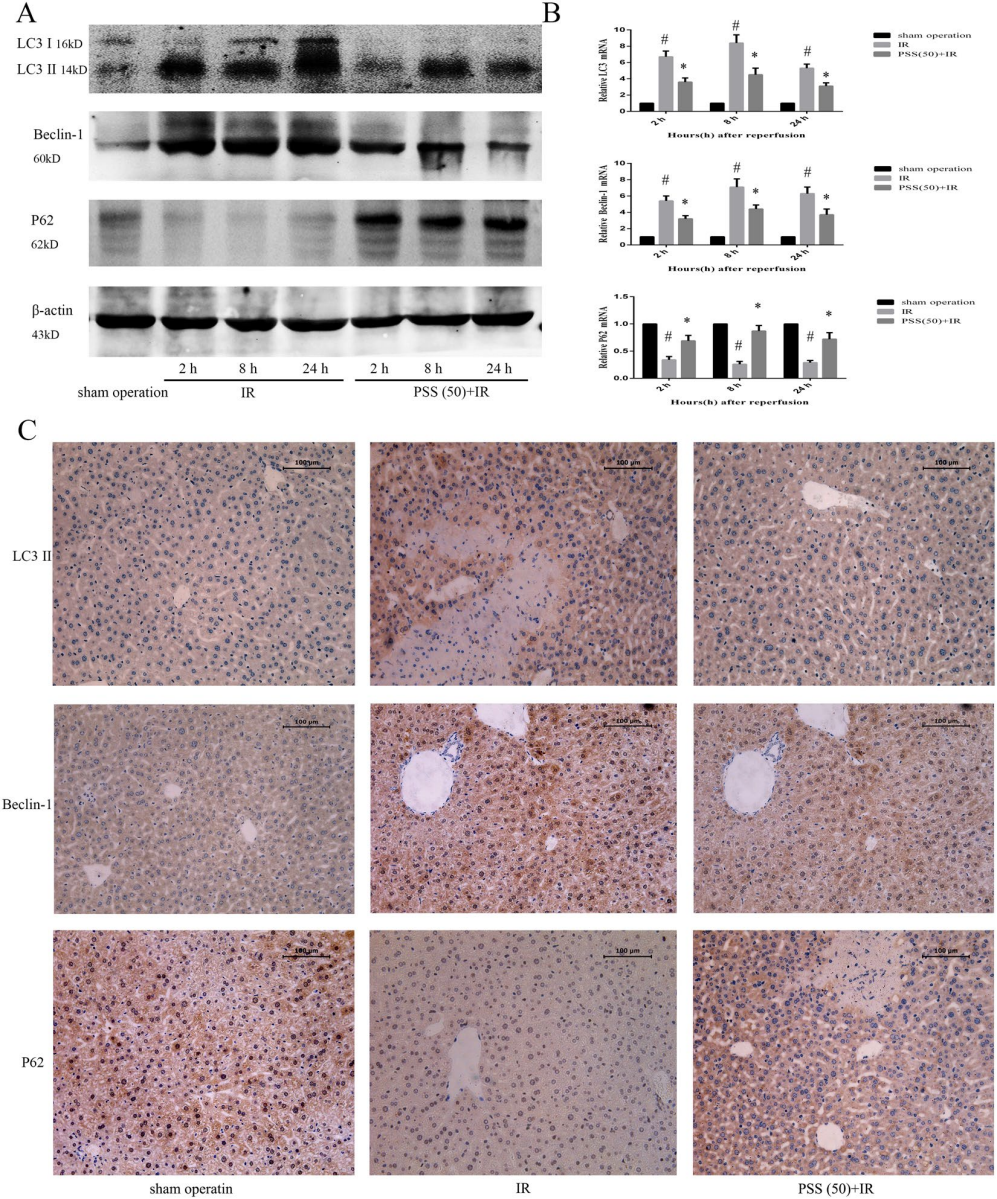
Anti-autophagy activity of PSS pretreatment in hepatic IR injury as shown by (**A**) expression of LC3 II, Beclin-1, and P62 protein assayed by western blotting. (**B**) expression of LC3 II, Beclin-1, and P62 mRNA by quantitative RT-PCR. Data expressed means ± SD (n = 6) ^#^P < 0.05 IR group vs. sham operation group, *P < 0.05 PSS (50) +IR group vs. IR group). (**C**) LC3 II, Beclin-1 and P62 immunostaining of liver sections from sham operation, IR, and PSS (50) +IR groups 8 h after IR. Representative sections of tissue from six mice (Original magnification, ×200).

Autophagy increased with IR injury, and was inhibited by PSS. Western blots and quantitative RT-PCR assays of autophagy-related proteins and their mRNAs revealed upregulation of LC3 and Beclin-1 in IR model mice and downregulation of P62, changes that were inhibited by PSS pretreatment (Fig. 5A & B). The results of immunohistochemical staining of LC3 and Beclin-1 were consistent with the western blot and RT-PCR results (Fig. 5C).

### PSS treatment prevented the MAPK pathway activation

**Figure 6:**
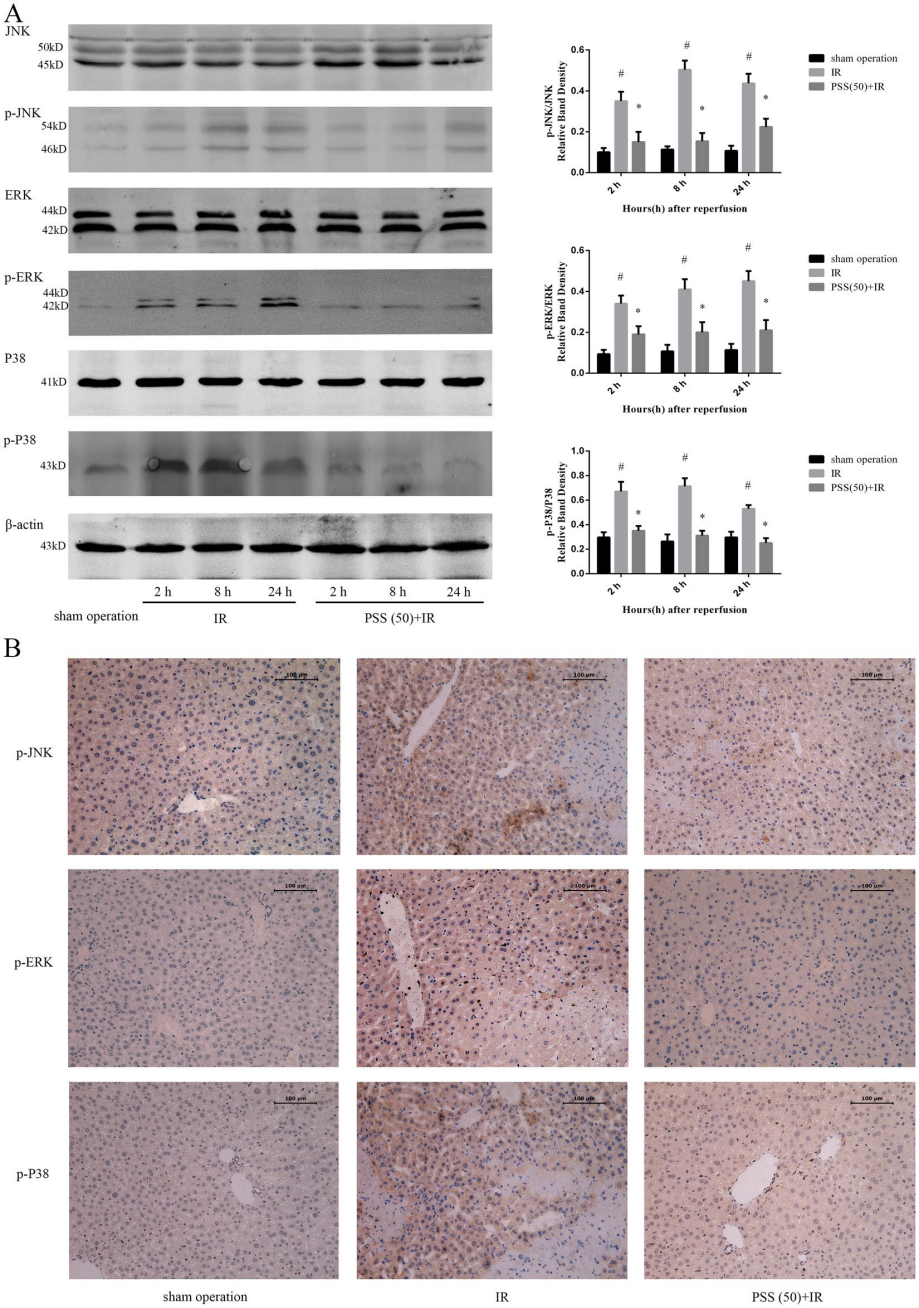
Effects of PSS treatment on MAPKs in hepatic IR injury as shown by (**A**) expression of total JNK, total ERK, total P38, phosphorylated JNK (p-JNK), phosphorylated ERK (p-ERK), phosphorylated P38 (p-P38) assayed by western blotting, (**B**) p-JNK, p-ERK, p-P38 immunostaining of liver sections from sham operation, IR, and PSS (50) +IR groups 8 h after IR. Representative sections of tissue from six mice (Original magnification, ×200).

As PSS treatment inhibited inflammation, apoptosis, and autophagy in liver tissue after IR, its effect on the MAPK pathway were investigated. Western blot assay of total and phosphorylated JNK, P38, and ERK expression found that PSS pretreatment significantly inhibited the activation of MAPKs that accompanied IR (Fig. 6A). Immunohistochemical staining confirmed those results (Fig. 6B).

## Discussion

Hepatic IR injury is a pathophysiological event that influences post-surgery outcome. In experimental animal models, pharmacological preconditioning has successfully managed hepatic IR injury^21^. PSS is a sulfated polysaccharide that contains β-D-mannuronic acid and α-L-guluronic acid that is prepared from brown seaweeds^22^. The anticoagulant, antihypertensive, and ability to reduce blood viscosity of PSS have been studied since 1986^4,23^. To the best of our knowledge, this is the first report of the protective functions of PSS against hepatic IR injury and the probable underlying molecular mechanism.

PSS pretreatment at both doses used, reduced the serum AST and ALT levels observed in the hepatic IR model mice without pretreatment. PSS pretreatment was also able to decrease the extent of hepatic necrosis, congestion, and edema produced by IR injury. The difference in the Suzuki criteria scores revealed PSS-related improvement in pathological changes characteristic of IR injury that were in line with the serological changes. The results consistently demonstrated that PSS pretreatment protected against hepatic IR injury.

The causes of hepatic IR injury are not clearly known, but acute inflammation is conducive to its development^24^. Kupffer cells are actively involved in warm IR injury, producing pro-inflammatory mediators, such as TNF-α, IL-6, and IFN-γ, which cause cellular injury^25,26^. This study assayed the expression of TNF-α, IL-6, and IFN-γ mRNA and protein as IR-induced inflammatory responses and found that increases of these cytokines after IR was prevented with PSS pretreatment. The results observed in expression of inflammatory markers were consistent with changes in serum AST and ALT and pathological changes in liver tissue. The results show that PSS pretreatment protected the liver by suppressing the release of inflammatory cytokines, including TNF-α, IL-6, and IFN-γ.

The MAPK signaling pathway is active in the progression of IR injury, and the involvement three of its family members, c-jun amino-terminal kinase (JNK), extracellular regulated kinases (ERK), and P38 MAPK in IR injury has been investigated^2,7,21^. Recent studies showed the production of inflammatory factors including TNF-α, and activity of reactive oxygen species (ROS) which initiated hepatic IR injury regulated the activation of the MAPKs^2,14^. In this mice model of hepatic IR injury, phosphorylation of JNK, ERK, and P38 increased with IR injury and PSS pretreatment decreased phosphorylation of all three. These observations show that PSS protects against hepatic IR injury by inhibit the activation of the MAPK signaling pathway.

Three cell death processes, necrosis, apoptosis, and autophagy are active in hepatic IR injury^27,28^, and necrosis had been discussed above. Activation of ERK and JNK kinases by TNF-α or IL-6 can phosphorylate Bcl-2, and the balance with Bax activity regulates caspase-dependent cellular apoptosis^29,30^. The TUNEL assay showed that PSS pretreatment downregulated apoptosis in liver tissue that was associated with IR injury. The changes in mRNA and protein expression of apoptosis-related factors, including Bax, Bcl-2, and cleaved caspase 3 provide strong evidence PSS inhibited apoptosis after IR, and it was also supported by immunohistochemistry results.

Autophagy is a conserved intracellular process and is involved in the pathogenesis of human diseases^31,32^. Activation of P38 can lead to the initiation of autophagy, in which the ubiquitin binding adapter P62 is active by binding to LC3^33^. P62 and LC3 regulate the formation of autophagosome, and the accumulation of autophagosomes leads to physiological dysfunction in incomplete autophagy^34^. Besides, Bcl-2 can bind to Beclin-1 and form Beclin-1/Bcl-2 complexes to regulate autophagy^35^. LC3 and Beclin-1 mRNA and protein expression was upregulated after IR, and PSS pretreatment had an inhibitory effect. P62 expression was suppressed by IR and promoted with PSS pretreatment. Immunohistochemical staining of LC3, Beclin-1 and P62 was consistent with the western blotting and quantitative RT-PCR results. The results show that PSS pretreatment inhibited IR injury-associated autophagy.

**Figure 7:**
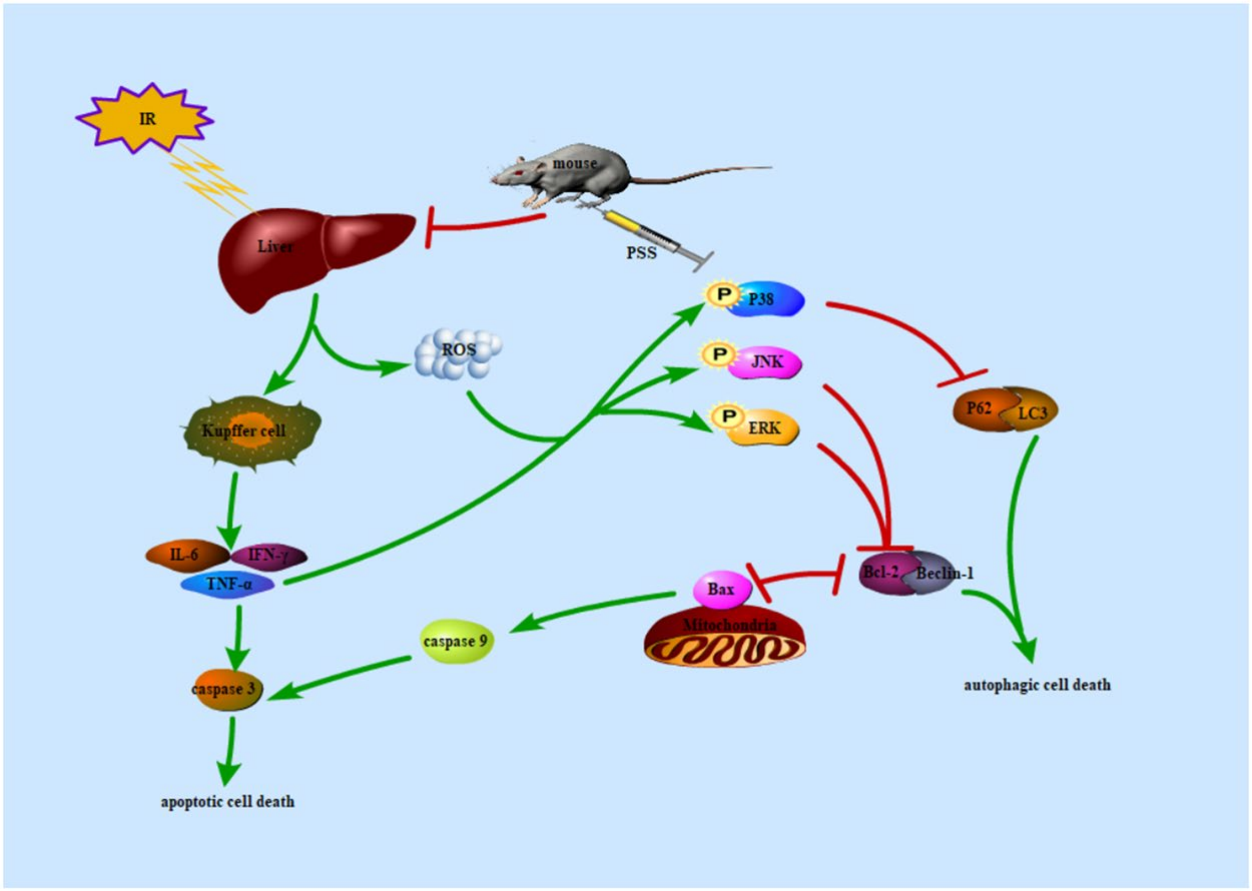
Probable mechanisms of PSS pretreatment in protecting against hepatic IR injury. Inflammatory cytokines including TNF-α, IL-6, and IFN-γ produced by Kupffer cells contribute to liver injury and upregulate caspase 3 to induce cellular apoptosis. Activation of JNK and ERK by phosphorylated Bcl-2, an anti-apoptotic protein, to facilitate the hepatocellular apoptosis and autophagy. The P38 MAPK activated by IR downregulates the P62 expression, which in turn leads to an increase of LC3 and formation of autophagosomes that promote autophagic cellular death. PSS treatment significantly downregulated the release of inflammatory factors and activation of MAPKs, reduced apoptosis and autophagy.

Despite many years of effort to prevent IR injury, its mechanisms are not well understood. PSS pretreatment had a consistent protective effect against hepatic IR injury because of its the anti-inflammatory, antiapoptotic and anti-autophagic activity. Activation of the MAPKs during IR injury were inhibited with PSS pretreatment and may underlying the preventive effect of PSS on hepatic IR injury (Fig. 7).

## Material and Methods

### Drugs and reagents

PSS with a molecular weight of 20.0 kDa was purchased from Dalian Tianyu Pharmaceuticals Co., Ltd (Dalian, China). ALT and AST microplate test kits were purchased from Nanjing Jiancheng Bioengineering Institute (Jiancheng Biotech, China). Enzyme-linked immunosorbent assay (ELISA) kits for TNF-α, IL-6, and IFN-γ measurement were purchased from Anogen-Yes Biotech (Mississauga, Canada). Anti-β-actin, TNF-α, IL-6, IFN-γ, Bax, Bcl-2, cleaved caspase 3, cleaved caspase 9, LC3, Beclin-1, P62, P38, ERK, JNK were from Proteintech (Chicago, IL, USA). Anti-p-P38, p-ERK, and p-JNK were from Cell Signaling Technology (Danvers, MA, USA). PCR assay kits were purchased from Takara Biotechnology (Dalian, China).

### Animals

Healthy male Balb/c mice weighting 23 ± 2 g were purchased at 6–8 weeks of age (Shanghai Laboratory Animal Co., Ltd., Shanghai, China) and kept in clean cages at 24 ± 2 °C and a 12 h light/dark cycle. The mice were provided with drinking water and food. All procedures involving mice followed the animal care protocols of the China National Institutes of Health and the Animal Care and Use Committee of Shanghai Tongji University, China. The study was approved by the Science and Technology Commission of Shanghai Municipality (ID: SYXK 2011-0111).

### Establishment of the hepatic IR model and experimental design

PSS (25 or 50 mg/kg) was dissolved in physiological saline solution and stored at 4 °C, in the dark until used. Prior to surgery and establishment of the IR injury model, the mice were randomly assigned to seven groups of 18 animals each as shown in Table 1.

**Table 1:** Group assignment and experimental procedures.

Experimental group (n = 18)	Treatment (intraperitoneal injection 1 h before surgery)
Normal control (NC)	Saline solution without laparotomy
PSS (25)	25 mg/kg PSS without laparotomy
PSS (50)	50 mg/kg PSS without laparotomy
Sham operation	Saline solution and surgical procedure without clamping
IR	Saline solution and all surgical procedures.
PSS (25) +IR	25 mg/kg PSS and all surgical procedures.
PSS (50) +IR	50 mg/kg PSS and all surgical procedures

Six mice in the NC, PSS (25) and PSS (50) groups were randomly anesthetized and sacrificed 24 h after treatment. Six mice in the sham operation, IR, PSS (25) +IR, and PSS (50) +IR groups were randomly anesthetized and sacrificed at 2, 8, and 24 h after reperfusion.

At sacrifice, blood samples were taken and centrifuged for 10 min at 4500 × g at 4 °C. Liver tissue samples were isolated, washed with saline solution, and immediately fixed in 10% formalin or frozen in liquid nitrogen. Serum and frozen tissue were stored at −80 °C.

A 70% hepatic warm ischemia model was established as previously described^35^. Mice were fasted for 24 h and then anesthetized by intraperitoneal injection of 1.25% sodium pentobarbital (Nembutal, St. Louis, MO, USA). The mice were immobilized and the liver was exposed with a median abdominal incision. The portal triad of the hepatic artery, portal vein, and bile duct were partially occluded with a microvascular clamp to produce 70% hepatic ischemia for 45 min, with tissue blanching as evidence of ischemia^37^. An animal body temperature maintenance instrument (ZS Dichuang, Beijing, China) was used to keep the body temperature constant. Reperfusion began with removal of the clamps and verified by an immediate color change of the ischemic liver lobes^37^.

### Serum analysis

Serum alanine aminotransferase (ALT) and aspartate aminotransferase (AST) levels were used as indicators of hepatocellular injury, and were assayed with an automated chemistry analyzer (Olympus AU1000, Tokyo, Japan). Serum TNF-α, IL-6, and IFN-γ were determined by enzyme-linked immunosorbent assay (ELISA) kits (Anogen-Yes Biotech, Mississauga, Canada) following the manufacturer’s instructions, and indicated the severity of inflammation caused by IR.

### Liver histology

Formalin-fixed liver tissue was dehydrated, embedded in paraffin. Serial 5 μm sections were cut and stained with hematoxylin and eosin (HE) for evaluation of hepatic necrosis. Five high-power (×200 magnification) fields were evaluated by light microscopy. The severity of hepatic IR injury was blindly graded using Suzuki’s criteria on a scale of 0 to 4 depending on the extent of sinusoidal congestion, hepatocyte necrosis, and ballooning degeneration. No necrosis, congestion, or centrilobular ballooning were given a score of 0. Severe congestion or ballooning degeneration, and lobular necrosis (more than 60%) were given a score of 4^38^.

### Immunohistochemistry

Paraffin-embedded liver sections were dewaxed and rehydrated. Antigen retrieval was performed by boiling in citric acid buffer and endogenous peroxidase activity was quenched with 3% hydrogen peroxide. Sections were then incubated with primary antibodies: TNF-α (1:100); IL-6 (1:100); IFN-γ (1:100); Bax (1:100); Bcl-2 (1:100); LC3 (1:200); P62 (1:100); p-P38 (1:400); p-ERK (1:200); and p-JNK (1:100) overnight at 4°C. Appropriate biotinylated secondary antibodies and a peroxidase substrate (DAB) kit (Vector, CA, USA) were used to visualize the primary antibodies. The sections were observed by a light microscopy and photographed with a digital camera (Leica Wetzlar, Germany).

### Terminal deoxynucleotidyl transferase-mediated deoxyuridine triphosphate nick-end labeling (TUNEL) assay

Paraffin-embedded sections were dewaxed and rehydrated, and TUNEL staining was performed following the manufacturer’s instructions. The observation and quantification of each specimen were performed in 10 high-power fields (×200 magnification), and cells with brown nuclei were identified as positive.

### Quantitative real-time polymerase chain reaction (RT-PCR)

Total RNA was extracted from approximately 100 mg of frozen liver tissue using TRIzol reagent (Takara, Shiga, Japan). After the RNA purity and concentration were detected, it was reverse transcribed using a ThermoScript RT-PCR system (Invitrogen). A 10 μL reaction system including primers, cDNA and the required enzymes was prepared for SYBR Green quantitative RT-PCR using a ViiA^TM^ 7 Real-Time PCR System (Applied Biosystems, Foster City, CA, USA). Gene expression was calculated as 2^-[(ΔCt of different groups)-(ΔCt of sham operation group)]^, in which ΔCt was the difference of the Ct values of the target gene transcript and the endogenous control β-actin. The results represented fold induction of different groups compared with baseline levels of sham operation groups. RT-PCR or all target genes was repeated three times. The primers used are shown in Table 2.

**Table 2:** Gene primers used in the RT-PCR assays.

Gene	GenBank Accession		Primers sequence (5′-3′)
β-actin	**NM_007393**	Forward	GGCTGTATTCCCCTCCATCG
		Reverse	CCAGTTGGTAACAATGCCATGT
TNF-α	**NM_000594**	Forward	CAGGCGGTGCCTATGTCTC
		Reverse	CGATCACCCCGAAGTTCAGTAG
IL-6	**NM_031168**	Forward	CTGCAAGAGACTTCCATCCAG
		Reverse	AGTGGTATAGACAGGTCTGTTGG
IFN-γ	**NM_008337**	Forward	GCCACGGCACAGTCATTGA
		Reverse	TGCTGATGGCCTGATTGTCTT
Bax	**NM_007527**	Forward	AGACAGGGGCCTTTTTGCTAC
		Reverse	AATTCGCCGGAGACACTCG
Bcl-2	**NM_177410**	Forward	GCTACCGTCGTCGTGACTTCGC
		Reverse	CCCCACCGAACTCAAAGAAGG
LC3	**NM_025735**	Forward	GACCGCTGTAAGGAGGTGC
		Reverse	AGAAGCCGAAGGTTTCTTGGG
P62	**NM_011018**	Forward	GAGGCACCCCGAAACATGG
		Reverse	ACTTATAGCGAGTTCCCACCA

### Western blot assay

Total protein was extracted from liver homogenates suspended in radioimmunoprecipitation assay lysis buffer containing protease and phosphatase inhibitors. Western blot assays were performed with aliquots containing 80 μg of total protein. Samples were electrophoresed on 10% or 12.5% sodium dodecyl sulfate polyacrylamide gels. Bands were blotted onto polyvinylidene fluoride (PVDF) membranes and incubated with primary antibodies: β-actin (1:2000); TNF-α (1:500); IL-6 (1:500); IFN-γ (1:500); Bax (1:1000); Bcl-2 (1:1000); cleaved caspase 3 (1:1000); cleaved caspase 9 (1:1000); LC3 (1:1000); Beclin-1 (1:1000); P62 (1:1000); P38 (1:1000); ERK (1:1000); JNK(1:1000); p-P38 (1:500); p-ERK (1:500); and p-JNK (1:500), as previously described^36^. Relative quantities of the different proteins were determined using an Odyssey two-color infrared laser imaging system (LI-COR Biosciences, Lincoln, NE, USA).

### Statistical analysis

All experiments were performed at least three times unless otherwise indicated. Results were expressed as means ± standard deviation (SD) and differences were analyzed by the Student *t*-test. A p-value < 0.05 was considered statistically significant.

### Data availability statement

The datasets generated during and/or analysed during the current study are available from the corresponding author on reasonable request.

## Conclusion

PSS treatment partly prevented hepatic IR injury by reducing inflammatory cytokines and apoptotic and autophagic cell death. The mechanism probably depended on suppression of the MAPKs. Therefore, PSS has potential clinical value for preventing hepatic IR injury.
